# Editorial: Drug re-purposing for the treatment of bacterial infections

**DOI:** 10.3389/fphar.2022.973730

**Published:** 2022-08-05

**Authors:** María Tomás, Younes Smani

**Affiliations:** ^1^ Microbiology Translational and Multidisciplinary (MicroTM)-Research Institute Biomedical A Coruña (INIBIC), Microbiology Deparment of Hospital A Coruña (CHUAC), University of A Coruña (UDC), A Coruña, Spain; ^2^ Department of Molecular Biology and Biochemical Engineering, Andalusian Center of Developmental Biology, CSIC, University of Pablo de Olavide, Seville, Spain

**Keywords:** repurposing, infections, MDR, bacteria, treatment

Bacterial infections are among the leading causes of death worldwide. The emergence of antimicrobial resistance factors threaten the efficacy of all current antimicrobial agents, with some already made ineffective, and, as a result, there is an urgent need for new treatment approaches. International organizations such as the World Health Organization (WHO) and the European Centre for Diseases Control (ECDC) have recognized infections caused by multidrug-resistant (MDR) bacteria as a priority for global health action, and new policies and actions are needed to avoid the predicted figures for 2050 of 10 million deaths caused by MDR bacteria.

Classical antimicrobial drug discovery involves *in vitro* screening for antimicrobial candidates, Structure Activity Relationship (SAR) analysis, followed by *in vivo* testing for toxicity. Bringing drugs from the bench to the bedside involves huge expenditures in time and resources. This, along with the relatively short window of therapeutic application for antibiotics attributed to the rapid emergence of drug resistance, has, at least until recently, resulted in a waning interest in antibiotic discovery among pharmaceutical companies. In this environment, “repurposing” (defined as investigating new uses for existing approved drugs) has gained renewed interest, as reflected by several recent studies, and may help to speed up the drug development process and save years of expensive research invested in antimicrobial drug development.

In this Research Topic focused on drug repurposing for the treatment of bacterial infections, four works (two original research articles and two reviews) were published on the use of herbal flavonoids, the optimization of antibiotic in monotherapy and in combination, and antibiotic adjuvants as repurposing drugs.

In reference to the optimization of antibiotics dosage in monotherapy and in combination Monte Carlo Simulations were performed. Lee et al. described the effect of extracorporeal membrane oxygenation (ECMO) on pharmacokinetic variability and target attainment of meropenem in a largest cohort of patients receiving ECMO. It is well-known that ECMO plays an important role in adult patients with heart or respiratory failure. During anti-infective treatment, altered pharmacokinetic patterns for meropenem caused by ECMO can affect therapeutic success. The authors of this study predicted through the Monte Carlo simulation the appropriate meropenem dosage regimen (1 g q8h by i. v. infusion over 0.5 h) for patients with ECMO and creatinine clearance of 50–130 ml/min (Lee et al.).

Yu et al. showed the activity comparison and optimal treatment of ceftazidime-avibactam and aztreonam-avibactam against bloodstream infections by carbapenem-resistant *Klebsiella pneumoniae* (CRKP) strains. The authors showed that in China, the ST11 KPC producing CRKP is major type, therefore, optimal management of ceftazidime-avibactam and aztreonam-avibactam against bloodstream infections by CRKP has important clinical significance to treatment. The Monte Carlo simulation performed in this study showed that the cumulative fraction of response of ceftazidime-avibactam and aztreonam-avibactam was 89% and 96%, respectively, using two-step infusion therapy (rapid first-step 0.5 h infusion and slow second-step 3 h infusion), 2 g q0.5 h infusion and 3 g q8 h with 3 h infusion for ceftazidime-avibactam, and 0.5 g with 0.5h and 1 g q8h for aztreonam-avibactam (Yu et al.).

In addition, Song et al. reviewed recent studies on antibacterial modes of herbal flavonoids against resistant bacteria, with a particular focus on the direct-acting and host-acting antibacterial flavonoids derived from heat-clearing herbs, to reveal the therapeutic strategies of resistant bacteria. The authors also outlined the availabilities and antibacterial pipelines of herbal flavonoids and discussed major challenges of flavonoids development, such as the screening of principles lead flavonoids based on the antibacterial target, the consideration of the multi-targets of flavonoids, and the guiding of the future drug modifications based on the structure and active antibacterial mechanism based on functional groups.

Finally, Boyd et al. provided a brief overview of approaches and challenges in new antibiotic development focusing on repurposing drugs. The authors have highlighted the antibiotic innovation gap discussing several combinations of factors such as the collapse of the Waksman platform, the golden age of medicinal chemistry, the adherence to Lipinski “rule of five”, the phenotypic versus target-based screens, and the high-risk investments. The authors also listed the drugs successfully repurposed in monotherapy or as an antibiotic adjuvant *in vitro* and *in vivo* against MDR bacteria and discussed the advantages of repurposing drug development.

In conclusion, repurposing drugs has gained interest and momentum in the last decade, as reflected in works published in this Research Topic of Frontiers in Pharmacology. Additional relevant Research Topic should be considered for the pre-clinical development of such compounds, including the use of animal models of infections for the assessment of their therapeutic efficacy, the determination of pharmacokinetic parameters, and the achievement of safety studies. The preclinical and clinical development of these approaches may help in the future to increase the arsenal of antibacterial drug families that can be used in monotherapy and/or in combination with antibiotics for the treatment of multidrug resistant bacterial infections.

